# Metformin as a potential combination therapy with existing front-line antibiotics for Tuberculosis

**DOI:** 10.1186/s12967-015-0443-y

**Published:** 2015-03-07

**Authors:** Rohit Vashisht, Samir K Brahmachari

**Affiliations:** Academy of Scientific and Innovative Research, New Delhi, India; CSIR-OSDD Unit, CSIR-IGIB, New Delhi, India; CSIR-Institute of Genomics and Integrative Biology, New Delhi, India

**Keywords:** Tuberculosis, Diabeties, Systems biology spindle map, Bacterial persistence, FDA approved drugs, Metformin

## Abstract

Tuberculosis (TB), the disease caused by Mycobacterium tuberculosis (Mtb) remains a global health concern. The evolution of various multi-drug resistant strains through genetic mutations or drug tolerant strains through bacterial persistence renders existing antibiotics ineffective. Hence there is need for the development of either new antibiotics or rationalizing approved drugs that can be utilized in combination with existing antibiotics as a therapeutic strategy. A comprehensive systems level mapping of metabolic complexity in Mtb revels a putative role of NDH-I in the formation of bacterial persistence under the influence of front-line antibiotics. Possibilities of targeting bacterial NDH-I with existing FDA approved drug for type-II diabetes, Metformin, along with existing front-line antibiotics is discussed and proposed as a potential combination therapy for TB.

Tuberculosis (TB) remains a leading global health concern with 5.4 million new cases reported in 2013 by WHO. Globally, 5% of TB cases are estimated to have Multi-Drug resistance TB (MDR-TB). The current anti-TB therapy constitutes a combination of 4 different antibiotics, which is extremely lengthy (6–9 months) and non compliance has resulted into the evolution of various MDR and extensively drug resistant strains of *Mycobacterium tuberculosis (Mtb)*, the main etiological agent of human tuberculosis. The problem of antibiotic resistance therefore renders current therapeutic interventions for the treatment of TB ineffective and hence urges the discovery or development of new and alternate strategies to counter its impact. Bacteria can develop antibiotic resistance either through genetic mutations [1] or by demonstrating bacterial persistence [2] when challenged with antibiotics. While the genetic bases of antibiotic resistance in *Mtb* are well established and understood, the aspect of bacterial persistence, which may result into drug tolerant phenotypes of *Mtb* is seldom addressed.

In our previous analysis, by utilizing a comprehensive *in silico* systems approach, we probed the metabolism in *Mtb* to identify various metabolic mechanisms that may potentiate the formation of persister phenotype in *Mtb* when challenged with current front-line antibiotics of TB therapy (Figure 1) [3]. Our analysis reveled directional re-routing of metabolic fluxes through NAD *de novo* biosynthesis pathway (encoded by *nadA ~ E* operon) and respiratory chain complex – I (NDH-I, encoded by *nuoA ~ N* operon) in *Mtb* as a possible alternate mechanism of ATP generation that may facilitate the formation of a persister phenotype and hence demonstrate antibiotic tolerance (Figure 1) [3].

**Figure 1:**
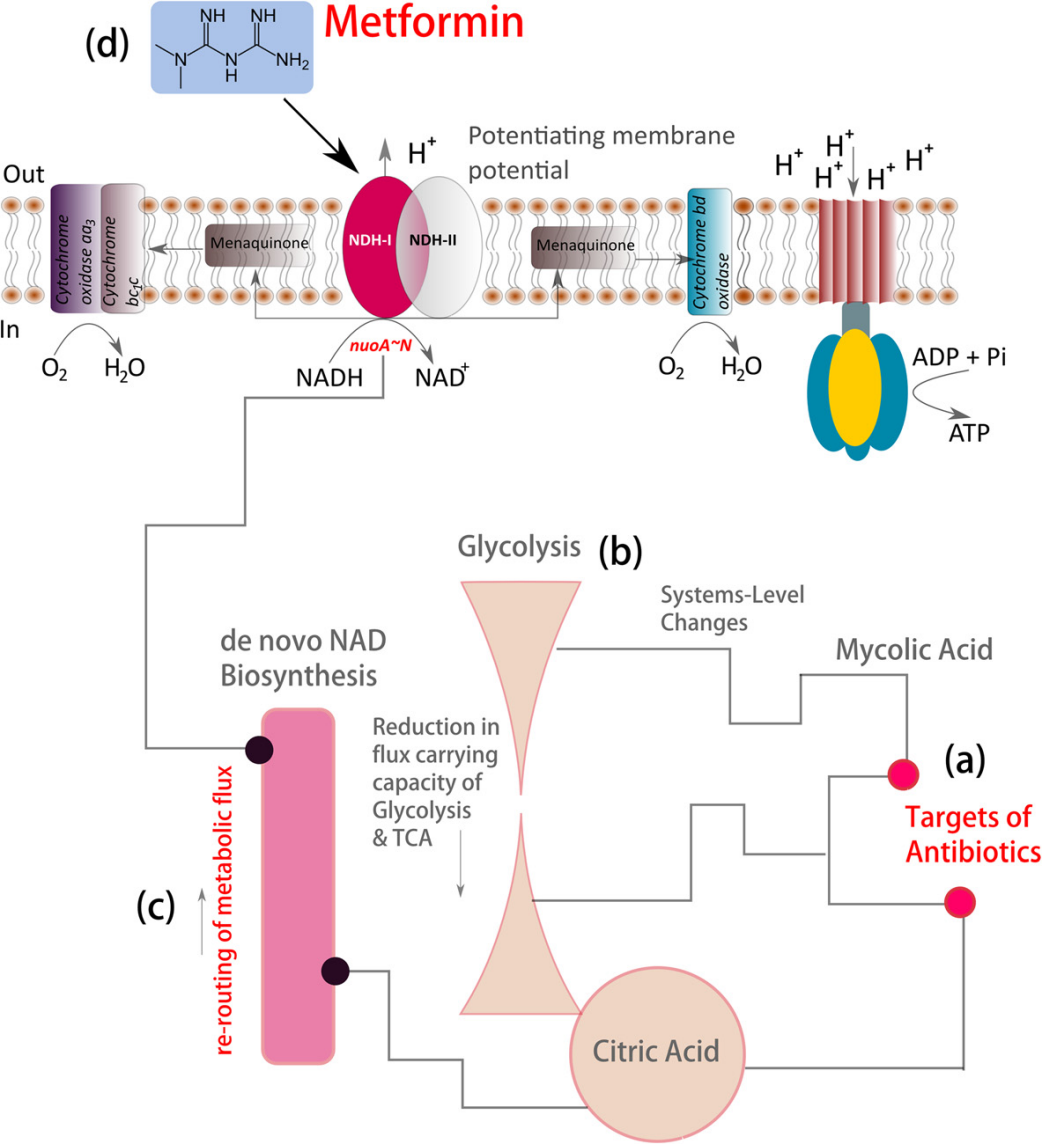
**Metformin, an FDA approved drug for type-II diabetes as a potential combination therapy for Tuberculosis with existing antibiotics. a)** Antibiotic targeting mycolic acid biosynthesis; **b)** systems-level changes resulting into the reduction of flux carrying capacity of glycolysis and citric acid cycle **c)** resulting re-routing of metabolic fluxes through de novo NAD biosynthesis pathway and electron transport through NDH-I **d)** possibility of targeting NDH-I with metformin.

Targeting proteins encoding for respiratory chain complex NDH-I and NAD *de novo* biosynthesis pathway in combination with targets of existing front-line antibiotics therefore becomes an intuitive strategy to counter the impact of drug tolerance mediated through bacterial persistence and hence can lead to the development of new therapeutic intervention for the treatment of TB.

Towards this we speculate that the Metformin, an inhibitor of mitochondrial complex-I, which is similar to bacterial NDH-I complex can be a potential drug candidate that can be utilized with existing antibiotics for targeting drug tolerant *Mtb* (Figure 1). Currently, Metformin is an FDA approved drug that is widely used for the treatment of patients with type-II diabetes [4]. The possibilities of utilizing Metformin as a combination drug with existing antibiotics for TB therapy are reasoned below:

1. The bacterial respiratory chain complex NADH-Q oxidoreductase is classified into three main subunits a) proton (H^+^) translocating subunit, H^+^ - NADH-Q oxidoreductase (designated as NDH-I); b) sodium (Na^+^) translocating subunit, Na^+^ - NADH-Q oxidoreductase (designated as Na^+^ - NADH) and c) NADH-Q oxidoreductase that lacks energy coupling site and designated as NDH-II [5].
2. Earlier investigations suggest that bacterial NDH-I is encoded by *nuoA ~ N* operon in *E.coli* [5], which is also encoded by *Mtb* genome [6].
3. A comparative analysis based on subunit sequence, cofactors and various inhibitors suggest that NDH-I of bacteria is the counter part of mitochondrial complex-I system [5].
4. Investigations also suggest that the bacterial NDH-I can be inhibited by various inhibitors of mitochondrial complex-I such as pericidin A, capsaicin and rolliniestain-1 thereby suggesting a common mechanism of NDH-I inhibition by inhibitors of mitochondrial complex – I [5].
5. Metformin has been recently confirmed as inhibitor of mitochondrial complex – I in cancer cells both *in vitro* and *in vivo*. Study also suggests implications of Metformin in reducing tumorigenesis [7].

Given the evidences that bacterial NDH-I complex is similar to mitochondrial complex – I in both its structure and function [5,6] and mitochondrial complex – I can be inhibited by Metformin [7], we therefore strongly believe that Metformin can be a potential candidate to inhibit NDH – I for *Mtb*. A recent study that appeared while preparing this manuscript reports a host-mediated response initiated by activation of AMPK when treated with Metformin as a mechanism leading to inhibition of *Mtb* growth, restriction in disease immunopathology and enhancement in the efficacy of conventional drugs [8]. While this study focused on the host-mediated response for *Mtb* eradication, it however remains to be tested if Metformin can directly bind to NDH-I of *Mtb* in the presence of front-line antibiotics to elicit a bactericidal effect on persister phenotypes of *Mtb.* Elucidating whether Metformin can directly bind to NDH-I or not would improve our understanding of its precise mode of action in prokaryotes and complement the on going efforts of identifying novel combination therapy for TB which might be effective for both drug susceptible and drug resistant/tolerant bacteria.

Furthermore, our previous systems level analysis also suggest a possible role of *nadA ~ E* operon (de novo biosynthesis pathway for NAD (Figure 1(c)) in the formation of persister phenotype in *Mtb* [3] mediated through directional re-routing of metabolic fluxes under the influence of front-line antibiotics. This makes NAD *de novo* biosynthesis pathway (encoded by *nadA ~ E* operon) an attractive combination target along with targets of existing front-line antibiotics for TB therapy. Designing new ligands or re-purposing the existing compounds that can inhibit *nadA ~ E* operon in *Mtb* in combination with front-line anti-TB antibiotics therefore might result into novel therapeutic interventions that can substantially aid in the ongoing efforts of TB translation research.
